# Hippocampal Functional Dynamics Are Clinically Implicated in Autoimmune Encephalitis With Faciobrachial Dystonic Seizures

**DOI:** 10.3389/fneur.2018.00736

**Published:** 2018-09-04

**Authors:** Julia C. Nantes, Adam G. Thomas, Natalie L. Voets, Jonathan G. Best, Clive R. Rosenthal, Adam Al-Diwani, Sarosh R. Irani, Charlotte J. Stagg

**Affiliations:** ^1^Physiological Neuroimaging Group, Wellcome Centre for Integrative Neuroimaging, FMRIB, Nuffield Department of Clinical Neurosciences, University of Oxford, Oxford, United Kingdom; ^2^Department of Neurology and Neurosurgery, McGill University, Montreal, QC, Canada; ^3^Section on Functional Imaging Methods, Department of Health and Human Services, National Institute of Mental Health, National Institutes of Health, Bethesda, MD, United States; ^4^Autoimmune Neurology Group, Nuffield Department of Clinical Neurosciences, Oxford, United Kingdom; ^5^Division of Clinical Neurology, Nuffield Department of Clinical Neurosciences, University of Oxford, Oxford, United Kingdom; ^6^Department of Psychiatry, University of Oxford, Oxford, United Kingdom; ^7^Department of Psychiatry, Oxford Centre for Human Brain Activity, Wellcome Centre for Integrative Neuroimaging, University of Oxford, Oxford, United Kingdom

**Keywords:** seizures, hippocampus, fMRI, LGI1, cognitive impairment

## Abstract

This is the first study to investigate functional brain activity in patients affected by autoimmune encephalitis with faciobrancial dystonic seizures (FBDS). Multimodal 3T MRI scans, including structural neuroimaging (T1-weighted, diffusion weighted) and functional neuroimaging (scene-encoding task known to activate hippocampal regions), were performed. This case series analysis included eight patients treated for autoimmune encephalitis with FBDS, scanned during the convalescent phase of their condition (median 1.1 years post-onset), and eight healthy volunteers. Compared to controls, 50% of patients showed abnormal hippocampal activity during scene-encoding relative to familiar scene-viewing. Higher peak FBDS frequency was significantly related to lower hippocampal activity during scene-encoding (*p* = 0.02), though not to markers of hippocampal microstructure (mean diffusivity, *p* = 0.3) or atrophy (normalized volume, *p* = 0.4). During scene-encoding, stronger within-medial temporal lobe (MTL) functional connectivity correlated with poorer Addenbrooke's Cognitive Examination-Revised memory score (*p* = 0.03). These findings suggest that in autoimmune encephalitis, frequent seizures may have a long-term impact on hippocampal activity, beyond that of structural damage. These observations also suggest a potential approach to determine on-going MTL performance in this condition to guide long-term management and future clinical trials.

## Introduction

Neuronal surface autoantibodies are likely causative in a variety of severe, yet often remarkably immunotherapy-responsive, neurological disorders involving epileptic activity (1). In recent years, faciobrachial dystonic seizures (FBDS), characterized by very frequent, short-lived seizures that typically affect the arm and face, have been identified as an early sign of certain forms of autoimmune encephalitis (2). FBDS are associated with autoantibodies directed at leucine-rich, glioma-inactivated 1 (LGI1) (2, 3) a secreted protein strongly expressed in key memory structures of the medial temporal lobe (MTL), including the hippocampus (4). LGI1 modulates synaptic transmission through interactions with presynaptic voltage-gated potassium channels (VGKC) and postsynaptic α-amino-3-hydroxy-5-methyl-4-isoxazolepropionic-acid (AMPA) receptors (5). More rarely, FBDS can be associated with alternative antigenic targets within the VGKC-complex (3).

Individuals with FBDS often develop cognitive impairment after seizure onset (2, 6). Both FBDS and the associated cognitive impairment appear to respond to immunotherapies; however, patients typically have residual cognitive impairment post-treatment, predominantly memory problems (7, 8). While these lasting deficits have been linked to persisting structural damage to the hippocampus (3, 8–10), the brain's functional dynamics have not yet been explored. Here, we investigated the hypothesis that functional characteristics of the MTL are related to clinical features of this condition following treatment for FBDS-associated autoimmune encephalitis. Understanding brain mechanisms underlying persistent memory impairment in the convalescent phase of this condition is a critically important step in directing the search for therapeutic options that may mitigate the long-term impact of autoantibody-associated epileptic activity.

## Materials and methods

Eight treated patients with a presenting clinical diagnosis of autoimmune encephalitis with FBDS participated (Table 1). Treatment of this patient cohort has been reported previously (3). The Addenbrooke's Cognitive Examination-Revised (ACE-R) was administered to patients during the acute phase and on the day of neuroimaging [years since onset: median = 1.1, quartiles_(lower,upper)_ = 0.8, 1.3]. Deficits on the ACER in these patients were dominantly in the memory domain (3). Eight age- and sex-matched healthy volunteers also participated. The protocol was approved by the University of Oxford Central Research Ethics Committee. All subjects gave written informed consent in accordance with the Declaration of Helsinki.

**Table 1:** Individual demographic and clinical characteristics of participants treated for autoimmune encephalitis with FBDS.

**Participant ID**	** 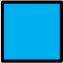 **	** 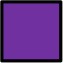 **	** 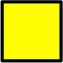 **	** 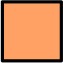 **	** 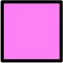 **	** 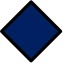 **	** 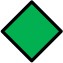 **	** 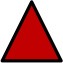 **
**DEMOGRAPHICS**								
Age range (years)	60–69	60–69	60–69	70–79	80–89	>90	40–49	70–79
**FBDS CHARACTERISTICS**								
Laterality	Bilateral	Bilateral	Bilateral	Bilateral (rapid alternating)	Bilateral (relapse) Right (originally)	Right	Right	Left
Affected body parts	Face Arm	Face Arm Leg	Face Arm Leg	Face Arm	Face Arm Leg	Face Arm	Face Arm	Face Arm
FBDS relapse	–	–	Yes	–	Yes	Yes	–	–
**OTHER FEATURES**								
Antigenic target(s)	LGI1	LGI1	LGI1 and CASPR2	LGI1	LGI1	LGI1	Unidentified VGKC complex protein	LGI1
Acute amnesia	Yes	–	Yes	Yes	Yes	Yes	–	–
Hippocampal volume (L/R)	3,752/3,930	3,783/3,435	3,485/3,551	3,166/3,373	2,632/2,613	1,941/2,857	4,171/3,743	3,460/3,296
Non-FBDS seizures	–	Yes	–	–	–	–	–	–
Time since onset (years)	0.6	5.3	1.8	1.1	1.0	1.1	0.3	0.8

Neuroimaging data were acquired on a 3T Siemens Vario scanner using a 32 channel head-coil. Due to technical reasons, data for one participant were acquired using a single channel receive head coil (blue diamond) (3). The protocol included: T1-weighted data using an Magnetization-Prepared Rapid Acquisition Gradient Echo sequence [1 × 1 × 1 mm resolution, TR = 2.53 s, inversion time 1.2 s, TE = 1.69, 3.55, 5.41, 7.27 ms, matrix size = 256 × 256 × 176, and Generalized Autocalibrating Partially Parallel Acquisition (GRAPPA) acceleration factor = 2], diffusion tensor-weighted echo-planar images (TE = 87 ms, TR = 9,600 ms, 65 slices, voxel size = 2 × 2 × 2 mm^3^, GRAPPA acceleration factor = 2, *b*-value of 1,000 s/mm^2^), and T2^*^-weighted Blood Oxygen Level Dependent (BOLD) echo planar images [TR = 2.41 s, TE = 30 ms, slice thickness = 3 mm, 44 slices, voxel size = 3 × 3 × 3 mm^3^, 128 volumes (first 3 removed)]. T2^*^-weighted BOLD echo-planar images [TR = 3.0 s, TE = 28 ms, slice thickness = 3 mm, 44 slices, voxel size 3 × 3 × 3 mm^3^, 183 volumes (first 2 removed)] were also acquired during performance of a *complex scene-encoding task* (11).

### Complex scene-encoding task

Immediately prior to the scan, subjects were shown four 30 s blocks of eight alternating complex color scenes (landscapes, buildings, or animals) (11, 12), which they were asked to memorize. The same eight images were shown in each block, though the order was randomized. During the subsequent fMRI scan, participants viewed a series of images over twelve 30 s blocks (eight images presented in each). These blocks, separated by 15 s visual fixation, alternated between displaying *familiar* scenes (i.e., the scenes participants were shown prior to the scan) in a random order each time, or viewing eight of the 48 *new* scenes (i.e., images they had not seen previously). Animals appeared in 4 of the 8 images (randomly ordered) in each block in both *familiar* and *new* conditions. To encourage encoding, participants were asked to indicate with button presses scenes that included animals and were told there would be a recognition test after the scan. Unfortunately, an insufficient number of participants completed the recognition test in a timely manner to enable the resulting data to be analyzed. Only 6/8 of subjects were able to complete the scene-encoding task, due to technical difficulties.

### Neuroimaging analyses

Eddy-current and motion-corrected mean diffusivity (MD) maps were created using FMRIB's Diffusion Toolbox and registered to T1-weighted images. Mean MD was then extracted from each hippocampus, defined using FreeSurfer-generated segmentations (https://surfer.nmr.mgh.harvard.edu/). FMRI data were preprocessed using FMRIB Software Library (FSL) (13–15) tools, including head motion correction, boundary-based registration to the T1-weighted image, and high-pass filtering (100 s).

**Figure 1:**
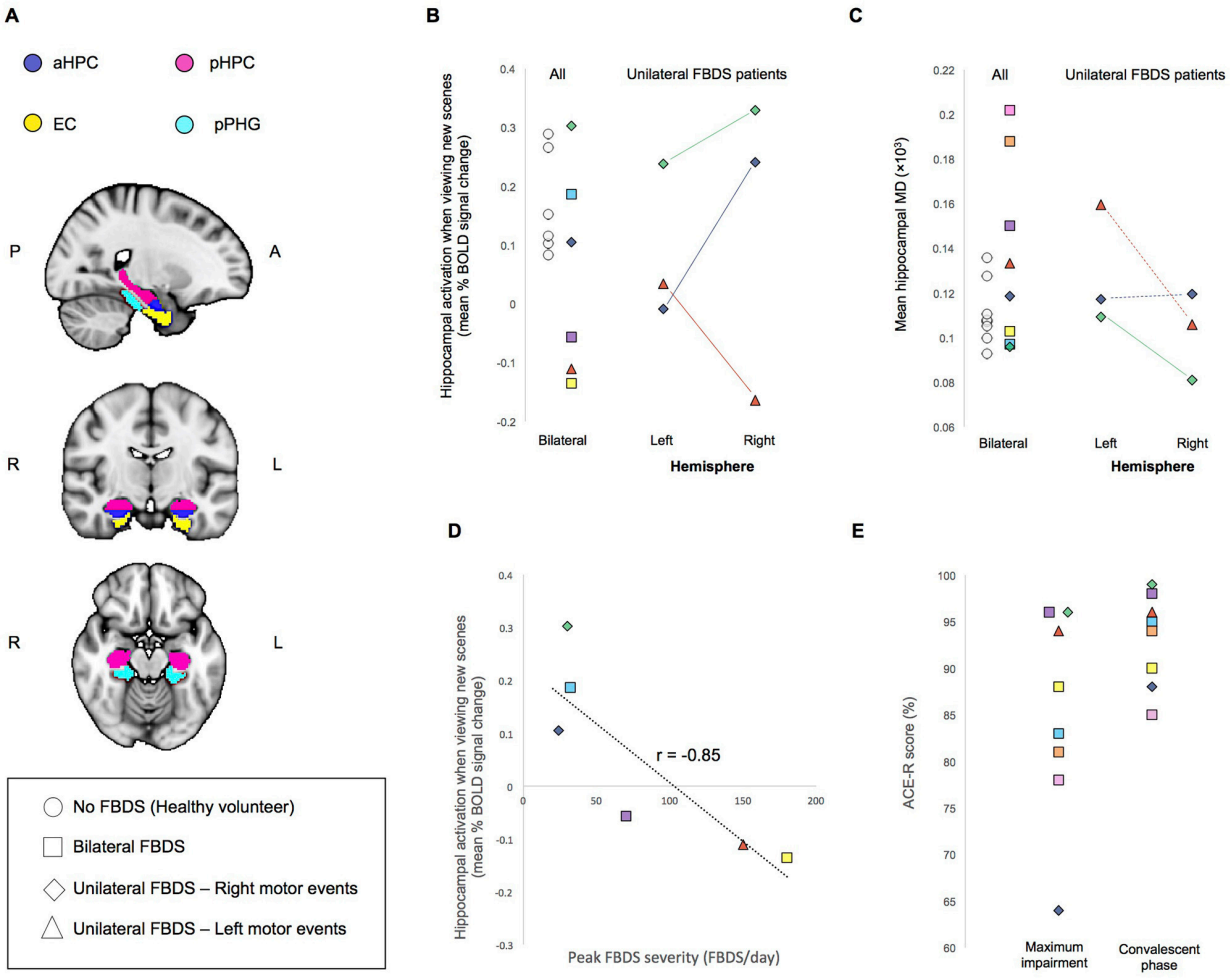
**(A)** Regions of Interest (ROIs) used for analysis. aHPC, anterior hippocampus; pHPC, posterior hippocampus; EC, entorhinal cortex; pPHG, posterior parahippocampal gyrus. **(B)** Mean percent BOLD signal difference during the encoding of new scenes verses viewing familiar scenes and **(C)** Mean hippocampal MD. For individuals who had unilateral FBDS, hemisphere specific measures are additionally plotted. Solid lines indicate between hemisphere differences in the predicted direction (based on clinically observed laterality of FBDS motor events), whereas dashed lines indicate between hemisphere differences were not in the predicted direction. **(D)** Correlation between peak FBDS severity and mean bilateral percent BOLD signal change in hippocampus during the encoding of new scenes compared to when viewing familiar scenes is plotted. Refer to Table 1 to match symbols of participating FBDS patients with individual demographic and clinical characteristics. **(E)** Peak ACE-R score at the time of peak impairment during the convalescent phase (day of neuroimaging assessment).

Medial temporal lobe regions of interest (ROIs) for resting-state and task-dependent fMRI analyses included the hippocampus, entorhinal cortex, and parahippocampal gyrus of each hemisphere (Figure 1A), which were derived from a previous study that used a different participant cohort (11). Mean percent signal changes, reflecting increased BOLD activity when viewing new scenes to encode compared to when viewing familiar scenes, were extracted at the single-subject level from the hippocampal ROI (11).

For functional connectivity analyses, we regressed out time-courses from the white matter and cerebrospinal fluid. Using the resting-state data, we investigated the strength of functional connections between hippocampal sub-regions and neighboring MTL cortical areas (entorhinal cortex, parahippocampal gyrus) previously linked to memory impairment in chronic forms of MTL epilepsy (11). Strength of anterior and posterior connections within each individual were averaged to create a single outcome metric reflecting overall functional connectivity between the hippocampus and neighboring MTL structures. We additionally assessed the strength of these connections with the task-dependent fMRI data, separately within blocks that included the presentation of *new* scenes to encode into memory and blocks during which participants viewed familiar scenes. This was to explore the existence of a psychophysiological change whereby these connections may be more relevant to memory while actively engaging in *complex scene-encoding* compared to in the resting-state or when viewing familiar scenes. We used a neuropsychological test of memory ability that was independent of the *complex scene-encoding task* when investigating clinical correlates of MTL functional dynamics.

### Statistical analyses

To investigate our primary questions of interest, regarding relationships between neuroimaging features of the MTL and clinical outcomes, correlational analyses (Pearson or Spearman rank analyses, where appropriate) and multiple linear regression analyses were performed using IMB SPSS Statistics software. As we hypothesized the direction of relationships assessed based on previous analyses on related clinical populations (9, 11), one-tailed statistical tests were used to determine significance. Multicollinearity and residuals were inspected for normality to confirm validity of general linear models. Non-parametric analyses were used for data that were not normally distributed. While this case series was not powered to statistically address the question of between-group differences in outcomes or the impact of seizure laterality, we explore the distribution of this data qualitatively.

## Results

### Qualitative case series data examination

Demographic and clinical characteristics are presented in Table 1. In three patients, FBDS remained strictly unilateral, and in another three patients no cognitive impairment was noted clinically during the acute illness (confirmed ACE-R > 94). None of the patients had abnormal MTL based on routine clinical imaging reports, and none had-ongoing seizures at the time of scanning.

Task-dependent hippocampal activity outcomes are presented in Figure 1B. Here, activity refers to mean within-ROI percent BOLD signal change when encoding new scenes relative to viewing familiar scenes. All healthy volunteers showed the expected positive mean bilateral percent signal change in the hippocampus during encoding. In contrast, only half of the participants who had autoimmune encephalitis demonstrated this normal activity pattern. Hippocampal MD was higher (indicating more damage) in 3/8 patients with FBDS than in any healthy volunteer (Figure 1C) and a statistical comparison of mean bilateral hippocampal MD between groups approached significance, indicating a trend toward elevated MD in this case series cohort of FBDS participants [*F*_(3, 16)_ = 2.9, *p* = 0.056].

Next, by focusing on the three participants with unilateral FBDS, we explored if seizure laterality might have influenced hemisphere-specific hippocampal activity or microstructure. Only one participant had hippocampal volume asymmetry [Table 1, reported previously (3)]. All three patients with unilateral FBDS had a between-hemisphere difference in functional activity in the hypothesized direction, with lower activity in the hippocampus contralateral to the side with FBDS motor events (Figure 1B), while consistent hemispheric differences in hippocampal MD were not observed (Figure 1C).

### Relationships between neuroimaging and clinical features

We investigated relationships between seizure burden and MTL function and found higher peak FBDS frequency was associated with lower mean bilateral hippocampal activity [Pearson correlation (*r*): *r*_(5)_ = −0.85, *p* = 0.02, Figure 1D]. By contrast, peak frequency of FBDS was not related to mean hippocampal MD [Spearman rank correlation (*r*_s_): *r*_s(5)_ = −0.25, *p* = 0.3] or hippocampal/total intracranial volume [*r*_s(5)_ = 0.06, *p* = 0.4]. Of note, patients were no longer experiencing clinical FBDS at the time of functional imaging, and ACE-R scores had improved significantly since the acute phase of the illness (Wilcoxon signed rank test: Z = 2.5, *p* = 0.006, Figure 1E). When including ACE-R score as a covariate in a linear regression analysis, peak frequency of FBDS remained significantly related to mean hippocampal activity (*r*_partial_ = −0.85, *p* = 0.04).

Next, we investigated functional connectivity between MTL structures of established hippocampal networks (11). Mean functional connectivity during the complex scene-encoding blocks (i.e., when presented with new images to memorize) between the hippocampus and neighboring MTL structures was significantly related to the ACE-R memory sub-score [*r*_(5)_ = −0.80, *p* = 0.03]. By contrast, these relationships did not reach significance during the resting-state scan (*p* = 0.3) or when participants viewed familiar scenes (*p* = 0.1). There was no relationship between duration of disease prior to immunotherapy and any of our clinical measures, though it is important to note that this can be a subjective measure.

Relationships between functional imaging and clinical features were maintained when excluding the one patient with VGKC-complex antibodies without LGI1 specificity, and, time between FBDS onset to neuroimaging was not significantly correlated with functional features of the MTL.

## Discussion

This is the first fMRI investigation, to our knowledge, on autoantibody-associated FBDS. Here, we sought to answer whether functional characteristics of the MTL were related to peak seizure severity and underpinned post-treatment residual memory impairment. This was an important issue to address considering that although patients treated for autoimmune encephalitis with FBDS improve acutely, their response to immunotherapies in the chronic phase of the illness course is often poor.

Participants performed a complex scene-encoding task that activates MTL structures bilaterally when instructed to remember new scenes compared to viewing familiar scenes (11). All healthy participants showed the expected (16) hippocampal activity in response to the novel images, whereas this was observed only for patients with a relatively low maximal frequency of FBDS. Indeed, high seizure frequency in the acute phase has recently been linked to longer-term clinical function impairments (1). Peak seizure frequency was, by contrast, not significantly linked to hippocampal volume or MD, suggesting that FBDS may have lasting functional consequences which are distinct from structural damage. Interestingly, the autoimmune encephalitis patient without LGI1 autoantibody specificity had relatively mild FBDS frequency at peak, was the only patient with a good response to antiepileptic drugs without immunotherapies (3), and, consistent with fMRI as a biomarker of severity, had normal functional activity in the hippocampus during the scene-encoding task.

While patients performed scene-encoding, stronger functional connectivity between anterior and posterior hippocampal regions and MTL cortical areas was linked to poorer performance on a fMRI task-independent test of memory performance (i.e., the ACE-R). This may suggest that inefficient functional connectivity within the MTL while actively engaged in memory processes is related to residual cognitive impairment post-acute treatment.

Limitations of our fMRI task included no interstimulus-interval jitter, meaning we were unable to investigate reaction times during task performance in the scanner, and inability to separate the effects of memory-encoding from stimulus novelty. Nonetheless, functional characteristics during blocks participants viewed novel scenes correlated with performance on a fMRI task-independent test of memory ability. The task performed was relatively simple, making it potentially practical for future clinical applications. While correlational outcomes of this case series investigation are robust, the sample size should be expanded in future studies. Investigations replicating these analyses and assessing other potentially clinically-relevant aspects of brain function in autoantibody-associated FBDS and related conditions are encouraged.

Although no patients were experiencing clinical FBDS at the time of scanning, the presence of subclinical seizure activity cannot be ruled out, which might affect both subjects' cognitive ability and the BOLD imaging results. Subclinical seizure activity is difficult to detect even with EEG; in the absence of electrophysiological recordings we cannot rule it out. Resting state approaches such as Arterial Spin Labeling (ASL) might be useful tools in future to identify increased resting activity levels which might be consistent with seizure activity, though the specificity of any changes would be difficult to determine.

## Conclusion

Functional characteristics of the MTL in patients treated for autoimmune encephalitis with FBDS may describe clinical features and parameters beyond that of structural damage, though the influence of subclinical seizure activity on these metrics cannot be ruled out. Future work may search to understand potential therapeutic modifiers of the brain's functional capacity to influence the long-term impact this condition. These functional imaging features may also serve as optimal biomarkers for treatment trials targeting seizures and chronic memory impairments.

## Author contributions

Design and conceptualization of the data acquisition protocol was performed by CS, SI, and NV. AT, NV, JB, CR, AA-D, and CS participated in data collection and study documentation. Neuroimaging and statistical data analyses were performed by JN, who also drafted the manuscript. All authors contributed to revising of the manuscript for intellectual content.

### Conflict of interest statement

The authors declare that the research was conducted in the absence of any commercial or financial relationships that could be construed as a potential conflict of interest.
